# Reviewer acknowledgement 2013

**DOI:** 10.1186/2047-2994-3-1

**Published:** 2014-01-16

**Authors:** Andreas Voss

**Affiliations:** 1 Canisius-Wilhelmina Hospital and Radboud University, Nijmegen Medical Centre, Netherlands

## Abstract

**Contributing reviewers:**

*Antimicrobial Resistance and Infection Control* would like to thank the following colleagues for their assistance with peer review of manuscripts for the journal in 2013.

## 

Michelle Alfa

Canada

Benedetta Allegranzi

Switzerland

Abdullah Assiri

Saudi Arabia

Marc Bonten

Netherlands

Malcolm Booth

United Kingdom

Jean Carlet

France

Mirian Dal Ben

Brazil

Olivier Denis

Belgium

Markus Dettenkofer

Germany

Philippe Eggimann

Switzerland

Juergen Gebel

Germany

Abdul Ghafur

India

Joost Hopman

Netherlands

Waleria Hryniewicz

Poland

Vincent Jarlier

France

Guenter Kampf

Germany

Hong Bin Kim

South Korea

Jan Kluytmans

Netherlands

Robin Köck

Germany

Axel Kramer

Germany

Andrea Kwa

Singapore

Sebastain Lemmen

Germany

François L’Hériteau

France

Moi Lin Ling

Singapore

John McGowan

United States of America

Mary-Louise McLaws

Australia

Geeta Mehta

India

Shaheen Mehtar

South Africa

Leonard Mermel

United States of America

Vivi Miriagou

Greece

Maria Luisa Moro

Italy

Matthew Muller

Canada

Torbjörn Noren

Sweden

Eric Nulens

Belgium

Angelo Pan

Italy

Yves Péan

France

Eli Perencevich

United States of America

Didier Pittet

Switzerland

Julie Robotham

United Kingdom

Phil Russo

Australia

Syed Sattar

Canada

Hugo Sax

Switzerland

Robert Skov

Denmark

Tom Sprong

Netherlands

Paul Tambyah

Singapore

Athanassios Tsakris

Greece

Josephus van Doornmalen

Netherlands

Erika Vlieghe

Belgium

Margreet Vos

Netherlands

Joanne Wai-yee Chung

Hong Kong

Guido Werner

Germany

Andreas Widmer

Switzerland

Reinhard Zbinden

Switzerland

Walter Zingg

Switzerland

